# Factors associated with the absence of postpartum consultations in a high-risk population

**DOI:** 10.61622/rbgo/2024rbgo23

**Published:** 2024-04-09

**Authors:** Ana Carolina Gomes Pereira, Tábata Regina Zumpano dos Santos, Helymar da Costa Machado, Fernanda Garanhani de Castro Surita

**Affiliations:** 1 Universidade Estadual de Campinas Campinas SP Brazil Universidade Estadual de Campinas, Campinas, SP, Brazil.

**Keywords:** Postpartum period, High-risk pregnancy, Referral and consultation, Postnatal care, Pregnant women

## Abstract

**Objective:**

To assess the rate of missed postpartum appointments at a referral center for high-risk pregnancy and compare puerperal women who did and did not attend these appointments to identify related factors.

**Methods:**

This was a retrospective cross-sectional study with all women scheduled for postpartum consultations at a high-risk obstetrics service in 2018. The variables selected to compare women were personal, obstetric, and perinatal. The variables of interest were obtained from the hospital's electronic medical records. Statistical analyses were performed using the Chi-square, Fisher's exact, or Mann–Whitney tests. For the variable of the interbirth interval, a receiver operating characteristic curve (ROC) was used to best discriminate whether or not patients attended the postpartum consultation. The significance level for the statistical tests was 5%.

**Results:**

A total of 1,629 women scheduled for postpartum consultations in 2018 were included. The rate of missing the postpartum consultation was 34.8%. A shorter interbirth interval (p = 0.039), previous use of psychoactive substances (p = 0.027), current or former smoking (p = 0.003), and multiparity (p < 0.001) were associated with non-attendance.

**Conclusion:**

This study showed a high rate of postpartum appointment non-attendance. This is particularly relevant because it was demonstrated in a high-risk obstetric service linked to clinical severity or social vulnerability cases. This highlights the need for new approaches to puerperal women before hospital discharge and new tools to increase adherence to postpartum consultations, especially for multiparous women.

## Introduction

The low utilization rate of postpartum medical consultations is a global health problem. Few studies have examined the puerperium, with most assessing women during antenatal care until childbirth. The Brazilian Ministry of Health *Guide on Women's Health During Pregnancy, Childbirth, and the Puerperium* recommends early assessment after discharge from the maternity hospital (ideally a home visit within 48 hours or a consultation at the Basic Health Unit in the first week after discharge), with a later evaluation between 30 and 40 days postpartum.^(1)^ The American College of Obstetricians and Gynecologists recommends a medical appointment within 3 weeks of childbirth, which should include a general assessment of physical and mental well-being, with particular emphasis on issues related to reproductive planning, breastfeeding assessment, and screening for postpartum depression.^(2)^

This postpartum follow-up is crucial to discuss the risks of complications and how they can influence the next pregnancy or the woman's life in the long term. At that time, for example, the woman should be given orientation about diabetes control and how to achieve an ideal weight in the period between pregnancies, which are important factors for maintaining health.^(3)^ Besides promoting women's health, more than half of pregnancy-related deaths occur after birth, which means that the postpartum period is not risk-free.^(4)^ For women with high-risk conditions and comorbidities, observing, monitoring, and facilitating post-pregnancy recovery may take several appointments.^(5)^ Future reproductive intentions provide the context for contraceptive decisions, which must be made in postpartum care.^(6)^ This is an opportunity not only to adjust the impact of subsequent pregnancy outcomes but also for family planning.^(7)^

Reproductive planning could prevent more than 30% of maternal deaths and 10% of child deaths if pregnancies are more than 2 years apart.^(8)^ The low utilization of postpartum care hinders not only access to contraception but also the treatment of possible pregnancy complications and chronic diseases. It also increases the risk of a new pregnancy in a short period. However, around 40% of postpartum consultations do not occur.^(9,10)^

Studies on women returning for postpartum care are scarce, especially in Brazil. However, a study obtained data regarding return rates for postpartum consultations, when associated with prenatal consultations. These data generally showed low coverage, with average values between 5% and 10% of women completing six prenatal consultations plus one postpartum consultation.^(11)^

Many important aspects of the postpartum period are still not well-explored. These include effective approaches to weight loss, when women can return to exercise, their return to work and how they deal with this, the main factors affecting mental and physical health, women's knowledge about the need for and relevance of breastfeeding, among many other topics needed to expand the knowledge about this population.^(2)^

To improve and expand the care provided to puerperal women, this study aimed to identify the socioeconomic, demographic, obstetric, and perinatal conditions that influence women, especially those in high-risk conditions, to not attend appointments. Thus, establishing strategies to change this situation will soon be possible.

## Methods

We performed a retrospective cross-sectional study at the Women's Hospital at the University of Campinas (UNICAMP), Brazil. This referral public university hospital in southeast Brazil serves a surrounding population of 3,100,000. We included all high-risk women scheduled for a postpartum consultation in this setting over 1 year (01/01/2018 to 12/31/2018).

In the outpatient clinic where this study was performed, the first postpartum consultation is scheduled around 6 weeks postpartum. However, for more severe cases, an earlier evaluation can be scheduled if necessary. A multidisciplinary team including doctors, nurses, psychologists, social workers, undergraduate students, and residents also takes turns to organize informative groups for new patients who do not know the service yet. Besides comprehensively evaluating the woman and requesting any necessary complementary tests and referrals, the woman chooses her contraceptive method, and the doctor explains the risks that the woman presents in the short and long term. The decision to discharge right after the first consultation or to follow up for some time is evaluated individually.

We selected variables to compare puerperal women who attended the consultation with those who did not. The sociodemographic variables considered were skin color, marital status, paid occupation, age, and place of origin (home); use of psychoactive substances (PAS), smoking, and alcohol; and previous or current diseases. The obstetric and perinatal variables considered were the interbirth interval, previous breastfeeding, obstetric risk, type of birth, how the labor began (induced or spontaneous), gestational age at birth, and Apgar score.

We collected all this information from the hospital's electronic medical records, and data were obtained from a typed form specifically created for the study in the EpiInfo 7® program. The data were entered into an Excel spreadsheet created for this study, which was reviewed to identify inconsistencies.

To describe the profile of the sample according to the variables under study, tables of categorical variables were created, with values of absolute frequency (n) and percentage (%), as well as descriptive statistics of numeric variables, with mean values, standard deviations, minimum and maximum values, medians, and quartiles.

To compare categorical variables between groups, we used the Chi-square or Fisher's exact tests (for expected values lower than 5). To compare numerical variables between groups, the Mann–Whitney test (two groups) was used due to the absence of normal distribution of variables. For the variable of the interbirth interval, an analysis was performed using the receiver operating characteristic (ROC) curve to find the cutoff point that best-discriminated patients who attended the postpartum consultation from those who did not. The significance level adopted for the statistical tests was 5%. The software used was the SAS System for Windows (Statistical Analysis System), version 9.2 (SAS Institute Inc, 2002–2008, Cary, NC, USA).

This study was approved by the Research Ethics Committee of the University of Campinas, Brazil (CAAE report 15420219.2.0000.5404). Due to the characteristics of the study, the consent form requirement was waived. All Strengthening the Reporting of Observational Studies in Epidemiology (STROBE) requirements for an observational study were followed and verified.^(12)^

## Results

In 2018, 1,629 women were scheduled for puerperal consultations, and 567 (34.8%) did not attend. Of the women included in the study, 970 (59.9%) lived in the same city where the hospital is located, 314 (19.3%) were teenagers (up to 19 years old), most were white (69.5%), and less than one third (27.5%) had a paid occupation. Table 1 shows the comparison of personal and sociodemographic data between women who did and did not attend the postpartum review appointment. The use of PAS and smoking was higher among women who did not attend the puerperal consultation.

**Table 1:** Comparison of sociodemographic variables between women who did and did not attend the postpartum appointment

Variables (n)		Missed the appointment n(%)	Attended the appointment n(%)	p-value
Skin color (1604)	White	379(68.4)	736(70.1)	0.486
	Non white	175(31.6)	314(29.9)	
Marital status (659)	Married	113(67.7)	304(61.8)	0.174
	Single	54(32.3)	188(38.2)	
Paid occupation (744)	No	125(69.4)	414(73.4)	0.301
	Yes	55(30.6)	150(26.6)	
Age in years (1629)	<19	115(20.3)	199(18.7)	0.192
	20-34	342(60.3)	616(58.0)	
	> 35	110(19.4)	247(23.3)	
Lives in Campinas*(1618)	Yes	338(60.0)	632(60.0)	0.990
	No	226(40.0)	422(40.0)	
Use of PAS**(745)	No	171(95.0)	555(98.2)	0.027
	Yes	9(5.0)	10(1.8)	
Smoking (745)	Yes	25(13.9)	40(7.0)	0.003
	No	135(75.0)	484(85.7)	
	Former smoker	20(11.1)	41(7.3)	
Alcoholism (745)	Yes	9(5.0)	16(2.8)	0.170
	No	161(89.4)	529(93.6)	
	Former drinker	10(5.6)	20(3.6)	
Previous or current diseases (244)	No	19(59.4)	98(46.2)	0.165
	Yes	13(40.6)	114(53.8)	

* Campinas: city where the hospital is located;

** PAS: psychoactive substances

Among women who attended the puerperal consultation (1,062), almost half (48.7%) had only one pregnancy, and 552 (67.5%) had 0–1 births, corresponding to primiparity or one previous abortion. Among the 226 primiparous women, who were going through their first postpartum experience, none missed the review appointment. In our total sample, 110 women (23.5%) had a history of two or more cesarean sections, and 24 (11.5%) had three or more previous abortions. Regarding the history of previous breastfeeding, 642 (86.3%) reported having breastfed previous children. More than half of the sample (127; 52.0%) had previous illnesses, and 783 (66.2%) had some obstetric risk. Among the obstetric risk factors presented were 72 cases (6.1%) of fetal malformations. Regarding the type of birth of the patient's last delivery (which motivated the scheduling of the postpartum appointment), 669 (56.5%) were cesarean sections. As for how the labor began, 421 (60.9%) occurred spontaneously. Regarding neonatal outcomes, 94 newborns (21.6%) were premature, with 25 (5.7%) born at less than 32 weeks of gestational age. Thirteen newborns (3.0%) had a 5-minute Apgar score of lower than 7. Table 2 shows the comparison of obstetric factors and neonatal outcomes between women who did and did not attend the postpartum consultation.

**Table 2:** Comparison of obstetric and neonatal variables between women who did and did not attend the postpartum appointment

Variables (n)		Missed the appointment n(%)	Attended the appointment n(%)	p-value
Interbirth interval in months (513)	Primiparous	0(0.0)	226(57.2)	<0.001
	< 12	2(1.7)	1(0.3)	
	13-24	16(13.5)	19(4.8)	
	> 25	100(84.8)	149(37.7)	
Interbirth interval in months excluding primiparous (513)	< 50	54(45.8)	52(30.8)	0.039
	> 51	64(54.2)	117(69.2)	
Previous breastfeeding (744)	No	21(11.7)	81(14.4)	0.360
	Yes	159(88.3)	483(85.6)	
Obstetric risk (1183)	No	138(36.8)	262(32.4)	0.139
	Yes	237(63.2)	546(67.6)	
Type of birth (1183)	Cesarean section	210(56.0)	459(56.8)	0.503
	Vaginal	165(44.0)	349(43.2)	
How labor started (691)	Spontaneous	145(65.0)	276(59.0)	0.128
	Induced	78(35.0)	192(41.0)	
Gestational age at birth in weeks (436)	< 32	5(3.5)	20(6.8)	0.130
	32 – 36	18(12.6)	51(17.4)	
	>= 37	120(83.9)	222(75.8)	
Apgar score (436)	< 7	5(3.6)	8(2.7)	0.764
	> 7	136(96.4)	287(97.3)	

**Figure 1:**
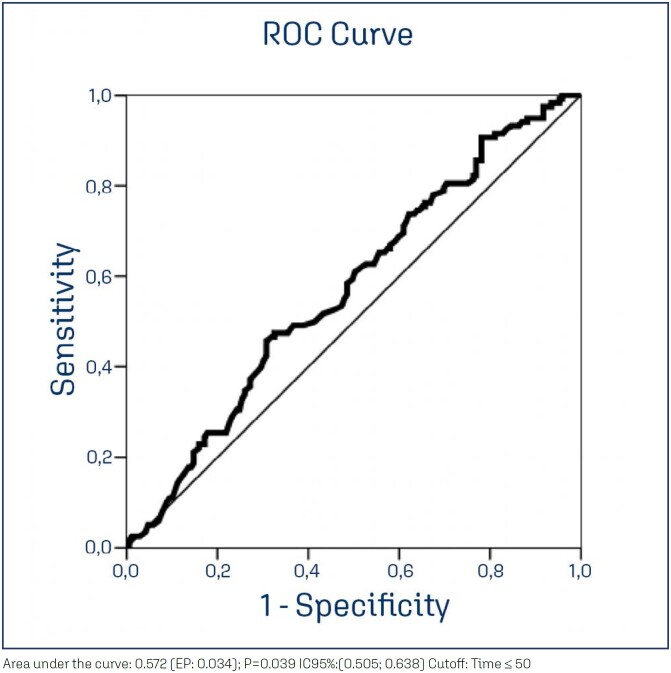
Interbirth interval

Figure 1 shows the ROC curve analysis excluding primiparous women, in which we see the cutoff point for interbirth interval as a predictor for postpartum absence. We found that the interval of ≤50 months obtained a significant area under the curve, associated with a greater probability of absence.

## Discussion

This study found a 34.8% rate of absence from the postpartum appointment. Despite the good functioning of the service, which is structured and involves a multidisciplinary team of well-trained and qualified professionals, the high rate of non-attendance is extremely relevant. This is especially so considering that the study was performed in a high-risk unit where women are linked to clinical severity or social vulnerability criteria.

According to international epidemiological data, health coverage for puerperal women is far from ideal. In the 2006 National Survey of Demography and Health of Children and Women, most women (60.8%) had no postpartum consultations.^(13)^ In another Brazilian study, the observed frequency of not attending the consultation was 24.8%.^(14)^ In Morocco, the rate was 21%, and in China, this proportion was 20.2%.^(15,16)^

The Brazilian public health system (Sistema Único de Saúde- SUS) advocates for the stratification of pregnant women according to their risk level (low, medium, or high). This determines the location for prenatal care: primary health care (PHC) centers for low and intermediate-risk and specialized ambulatory care (SAC) for high-risk cases. High-risk pregnant women—and subsequently high-risk postpartum women—receiving care at the SAC should have their care shared with PHC centers. In practice, a minority of patients maintain this connection.

Women who had one or more previous deliveries (multiparous), those with a shorter interbirth interval (less than 50 months), and users of tobacco and other PAS were more likely to not attend the postpartum consultation compared to primiparous women, those with an interbirth interval longer than 50 months, and those not using tobacco and other PAS.

We identified that 57.2% of the women who attended the postpartum consultation were primiparous; no primiparous women missed the appointment. The comparison showed statistical significance for the attendance of primiparous women when compared to women with more than one child. This may be because first pregnancies bring greater anxiety and more doubts regarding the first postpartum experience. Additionally, logistics and movement are easier for women with only one baby. Women with more than one child need a stronger support network to be able to leave their house to attend the hospital, often needing someone else to care for their other children. Unfortunately, this is a reality for many women in Brazil and other countries.^(9,10)^ The availability to return is strongly correlated with the conditions of the postpartum woman and her possibility of traveling with a newborn to the hospital.^(9,10)^

In Portugal, more barriers existed for women to attend postpartum consultations when they were younger, less educated, unemployed; had a lower income; and had medical complications during pregnancy.^(17)^ These data are similar to another study in Brazil, in which women with lower income, less education, some morbidity during pregnancy, and no contraception were least likely to return for follow-up.^(14)^

Even though most sexually active women use contraceptives, more than half of pregnancies in Brazil are unplanned. A possible explanation for this is that the most used methods in Latin America are short-duration, depending on the user to guarantee their effectiveness, which leads to more failures.^(18)^ Brazilian women in the first six postpartum months mainly have access to short-acting contraceptives, which are not what they would choose when asked about postpartum contraceptive methods while pregnant. Only 28.9% of all women can obtain the method they want.^(19)^

In countries with social inequality, wealthier women tend to use long-acting reversible contraceptives (LARCs) more than poor women do, even though LARCs are appropriate for most.^(18)^ This highlights the importance of medical consultation to promote information and ensure the possibility of choosing among the most effective methods for all women, specifically including highly efficient and long-lasting methods such as LARCs.

Women who do not use contraception, and those who are less present at puerperal consultations, are also the most exposed to recurrent and earlier pregnancies, tending to have a shorter and less adequate interbirth interval.^(14)^ This profile of women showing more absence confirms the findings of our current research, in which we observed that up to the interbirth interval cutoff of 4 years, women are more absent.

An interbirth interval of up to 24 months is considered short and inadequate, bringing maternal and fetal risks, especially below 18 and 12 months. Progressive worsening of perinatal results is associated with shorter intervals. Importantly, women with some morbidity during pregnancy were at greater risk of not having a postpartum consultation when compared to healthy women.^(14)^ Therefore, women with lower interbirth intervals are likely to face more logistical and mobility barriers due to their higher risk of morbidity or the number of young children.

We found a relationship between the greater absence of multiparous women and a shorter interbirth interval with no postpartum consultations. We thus emphasize the benefits and importance of postpartum reproductive planning, which include reducing the risk of premature birth, abortion, neonatal death, and maternal death.^(20)^

The use of PAS and current or previous smoking were also related to non-attendance at the postpartum appointment. The lower the woman's income and education, the greater the risk of not having a postpartum appointment.^(14)^ This is in line with the findings of this study that low economic class and lower education level are risk factors for smoking in Brazil.^(21)^

At one extreme are women with more years of study, the possibility of choosing their contraceptive methods, and fewer children. On the other hand, women with less education and fewer opportunities have more children—in most cases, from unplanned pregnancies.^(22)^ We can identify a certain profile of women who return less to appointments: those with lower incomes and lower education, who are more exposed to recurrent pregnancy, and consequently are multiparous.

A limitation of the study was the loss of some number of patients at most variables because the data used already existed in the hospital's computerized information system. The medical records were not complete for some patients, and some analyses had a sample with a smaller total number. This was because the study has been carried out for some time and the system has evolved during this period. The study also contributed to the improvement of the system. Previously, the system did not require mandatory fields to finalize postpartum appointments. Now, to close and finalize the consultation, all fields are required, and the database has become more complete. Another limitation is that women who miss appointments at the SAC (which was the case in the location where the study was conducted) should be followed up by a PHC center, but we did not have this data.

We are far from reaching the goal of a maximum of 30 maternal deaths per 100,000 live births, which is one of the UN Sustainable Development Goals for 2030. Home visits, health education, and referrals to specialized care are all actions that can be performed to reduce the maternal mortality rate in Brazil. The difference between the theory of public health politics and the applicability of those in practice remains a challenge for reducing maternal mortality. This situation may be related to the lack of infrastructure and resources to provide women's health care.^(23)^ The current proposal from the World Health Organization (WHO) is that healthy women and newborns should receive postnatal care in the facility for at least 24 hours, and at least three additional postnatal contacts are recommended for healthy women and newborns: between 48 and 72 hours, between 7 and 14 days, and during week 6 after birth. In other words, the WHO suggests earlier and more frequent appointments, given the importance of the puerperal period to maternal and child health.^(24)^ The four meetings suggested by the WHO may be enough for pregnant women at usual risk, but for women with high-risk conditions and comorbidities, even more consultations may be necessary for the professional to manage certain health conditions.^(25)^

A woman may not return to the postpartum consultation for many possible reasons. Talking about this during antenatal care and even during hospitalization is thus extremely important. Guidance about breastfeeding and possible symptoms of the puerperium has a positive effect on well-being. In a randomized clinical trial, 15 minutes of orientation before hospital discharge, followed by a follow-up call 2 weeks after discharge, leading to a reduction in symptoms of postpartum depression and an increase in the duration of breastfeeding up to 6 months postpartum among Black and Latino women in the United States.^(26)^

Strategies must be designed to increase attendance at postpartum appointments. These may include discussing the importance of postpartum care during antenatal care, scheduling postpartum visits before hospital discharge, and relying on technology (email, text, and apps) to remind women to attend postpartum follow-ups. Online care in the postpartum period is possible for women who cannot go to the hospital.

Efforts should be raised to reduce social inequalities in Brazil, increasing access to paid family leave and investing in family planning, especially with LARC methods and preferably before hospital discharge. The immediate postpartum insertion of intrauterine devices and subcutaneous implants is already being performed and generating good results in the hospital where this study was carried out.^(27)^ This is important to guarantee that even if a puerperal woman misses the appointment, she will be assured of her long-term contraception and the possibility of a more adequate interbirth interval.

## Conclusion

This study evaluated the rate of postpartum consultation nonattendance and its associated factors in a referral service for high-risk women. This rate was 34.8%, which is particularly relevant because it was demonstrated in a high-risk obstetric care service where the cases involve clinical severity or social vulnerability. Having more than one child, having an interbirth interval of fewer than 50 months, and using tobacco or PAS increased the chances of missing appointments among women in this study. This highlights the need for new approaches for puerperal women in antenatal care and before hospital discharge. New tools must be developed to increase adherence to puerperal review consultations, especially for women with more than one child.
